# *Iroquois Complex* Genes Induce Co-Expression of *rhodopsins* in *Drosophila*

**DOI:** 10.1371/journal.pbio.0060097

**Published:** 2008-04-22

**Authors:** Esteban O Mazzoni, Arzu Celik, Mathias F Wernet, Daniel Vasiliauskas, Robert J Johnston, Tiffany A Cook, Franck Pichaud, Claude Desplan

**Affiliations:** Center for Developmental Genetics, Department of Biology, New York University, New York, New York, United States of America; Purdue University, United States of America

## Abstract

The *Drosophila* eye is a mosaic that results from the stochastic distribution of two ommatidial subtypes. Pale and yellow ommatidia can be distinguished by the expression of distinct *rhodopsins* and other pigments in their inner photoreceptors (R7 and R8), which are implicated in color vision. The pale subtype contains ultraviolet (UV)-absorbing Rh3 in R7 and blue-absorbing Rh5 in R8. The yellow subtype contains UV-absorbing Rh4 in R7 and green-absorbing Rh6 in R8*.* The exclusive expression of one rhodopsin per photoreceptor is a widespread phenomenon, although exceptions exist. The mechanisms leading to the exclusive expression or to co-expression of sensory receptors are currently not known. We describe a new class of ommatidia that co-express *rh3* and *rh4* in R7, but maintain normal exclusion between *rh5* and *rh6* in R8. These ommatidia, which are localized in the dorsal eye, result from the expansion of *rh3* into the yellow-R7 subtype. Genes from the *Iroquois Complex* (*Iro-C*) are necessary and sufficient to induce co-expression in yR7. *Iro-C* genes allow photoreceptors to break the “one receptor–one neuron” rule, leading to a novel subtype of broad-spectrum UV- and green-sensitive ommatidia.

## Author Summary

Most sensory systems follow the rule “one receptor molecule per receptor cell.” For example, photoreceptors in the fly eye and cones in the human eye each express only one light-sensitive *rhodopsin*. Rhodopsins are G-coupled protein receptors, a class of ancient signaling molecules that mediate not just vision but also the sense of smell, the inflammatory response, and other physiological processes. However, the mechanisms that regulate mutual exclusion of receptor genes in the visual and olfactory systems are poorly understood. Each ommatidium in the fly eye consists of eight photoreceptors (R1–R8); six of which mediate broad-spectrum motion vision (R1–R6) and two that mediate color vision (R7 and R8). We identified a new class of photoreceptors in the fly retina that violates the one rhodopsin–one receptor rule. This subset of ommatidia, located in the dorsal third of the eye, co-expresses two ultraviolet-sensitive *rhodospins* (*rh3* and *rh4*) in R7, while maintaining discrimination between green and blue opsins in R8. We took advantage of the genetic tools offered by the fruit fly to show that this co-expression depends on the *Iroquois Complex (Iro-C*) genes that are both necessary and sufficient to allow the two ultraviolet-sensitive rhosopsins to be expressed in the same R7 cell. These results shed new light on the mechanisms regulating co-expression of rhodopsins in the eye, and may well have implications for regulating co-expression in olfactory receptors and other G-protein coupled systems.

## Abstract 3

*Iro-C* genes control the co-expression of sensory receptors.

## Introduction

The primary role of sensory organs is to probe the environment and to transmit precisely this information to the brain for processing. The visual and olfactory systems are composed of sensory epithelia with thousands of sensory receptor cells, each specifically expressing a single sensory receptor gene out of a much larger repertoire [1–6]. This “one receptor–one neuron” rule allows specific detection of sensory information at the periphery. Together, the architecture of the visual or olfactory organs, the correct specification of the sensory neurons, and the expression of specific sensory receptor molecules are crucial for the acquisition of sensory information. Sensory organs have thus adapted for optimal detection of specific stimuli and often exhibit spatial regionalization within the sensory organ itself. This regionalization also extends into topographic maps in the brain (retinotopy of the visual system, chemotopy in the olfactory system) [7].

The *Drosophila* compound eye is composed of approximately 750 simple eyes called ommatidia. Each ommatidium contains eight photoreceptor cells named R1–R8. The light-gathering structures (rhabdomeres) of outer photoreceptors (R1–R6) form an asymmetric trapezoid whose center is occupied by the rhabdomeres of the inner photoreceptors, where the R7 rhabdomere sits on top of that of R8 [8]. The last step in photoreceptor differentiation is the selective expression of one of the photosensitive pigments, the *rhodopsins*. The expression of a given *rhodopsin*, along with additional filtering or sensitizing pigments, dictates the color sensitivity of a photoreceptor. Five *rhodopsins* are expressed in the compound eye. They respect the general rule of “one receptor–one neuron”— R1–R6 cells express Rh1 [2]. The rhodopsins are similar in function to the vertebrate rods in that they are sensitive to a broad range of wavelengths. They are involved in motion detection. Inner photoreceptors (R7 and R8) mediate color vision [9,10], and are thus comparable to vertebrate cones [11,12]. These photoreceptors express the remaining four *rhodopsins*, which have a restricted spectrum of absorption ranging from ultraviolet (UV) in R7 to blue or green in R8 [1,3,4,13–15].

Although the eye appears to be composed of morphologically identical ommatidia, the main part of the retina consists of a mosaic of two stochastically distributed subtypes of ommatidia: pale type (p) contains a UV-absorbing Rh3 in R7 and a blue-absorbing Rh5 in R8; yellow type (y) contains a different UV-absorbing Rh4 in R7 and green-absorbing Rh6 in R8 [1,14,16]. A filtering pigment, “yellow” sharpens the sensitivity of yR7 and filters out the blue light reaching the green-sensitive underlying yR8 [17,18]. y ommatidia represent ∼70% of ommatidia in flies ranging from *Musca* to *Drosophila*. These ommatidia can now be defined more accurately based on their Rh content. The *Drosophila* homolog of the vertebrate dioxin receptor *spineless* (*ss*) is responsible for the specification of the retinal mosaic [19]. *ss* expression in ∼70% of R7 cells in pupae commits them to the yR7 fate and to express *rh4*. The cells that do not express *ss* become pR7, express *rh3*, and instruct pR8 to express *rh5*. By default, the remaining yR8 express *rh6* [1,20]*.* Thus, 30% of ommatidia (p) appear to be more involved in the discrimination of shorter wavelengths, whereas the remaining 70% (y) should be more appropriate for the discrimination of longer wavelengths.

The p and y ommatidia appear to be randomly distributed. The *Drosophila* eye, like in many insects, has also developed a particularly striking example of sensory system specialization in the dorsal rim area (DRA). DRA ommatidia develop in the dorsal-most row of the eye and have distinct morphological characteristics that enable them to be used to detect the electric vector (e-vector) of light polarization [21,22]. Because polarized light comes from UV-rich sunlight scattered by the atmosphere, this row of ommatidia is limited to the dorsal edge of the eye and must therefore be specified by positional cues [22,23].

Regionalization of tissues often starts very early during organogenesis and often involves conserved molecular mechanisms that are important for patterning tissues as different as *Drosophila* sensory systems or vertebrate limb buds. In the *Drosophila* eye imaginal disc, dorso-ventral compartmentalization involves the differential expression of genes of the *Iroquois Complex* (*Iro-C*). *Iro-C* genes encode conserved homeodomain transcription factors from the TALE class [24]—*araucan* (*ara*), *caupolican* (*caup*), and *mirror* (*mirr*)—and their genomic organization as a cluster of three genes is conserved from flies to mammals [25,26]. In *Drosophila*, *ara* and *caup* have almost identical patterns of expression [27], whereas *mirr* is more divergent. Among other functions, these three genes have been implicated in very early stages of eye-antennal disc development as “dorsal selectors” that are required for the correct specification of dorsal head structures and for the formation of the dorsal compartment of the eye [28–30]. During larval development, the *Iro-C* genes are expressed in dorsal nondifferentiated cells of the eye imaginal disc and are then down-regulated once neurogenesis has begun. This expression distinguishes different cell fates on either side of the dorso-ventral boundary and is necessary to establish the organizer center at the equator (reviewed in [26]). Although expression of *Iro-C* genes fades away after the morphogenetic furrow, their expression reappears in the adult. *Iro-C* genes are necessary to specify the DRA: ommatidia near the edge of the disc are exposed to *wingless* signaling and become DRA ommatidia only when they are located dorsally [22,23].

Here we describe a new function for *Iro-C* genes in photoreceptor development: they define a subtype of ommatidia that is restricted to the dorsal region of the eye in which the “one receptor–one neuron” rule is broken. These ommatidia are positioned in the dorsal part of the retina and co-express the two genes encoding UV-absorbing Rhs—*rh3* and *rh4*—in R7 cells. This co-expression results from the induction of *rh3* in yR7 cells while pR7 are normal. Therefore, the mutual exclusion pathway that prevents co-expression of sensory receptors appears to be disabled by the activity of the *Iro-C* genes, allowing the expression of two sensory receptors in a single cell.

## Results

### A Novel Class of Dorsal Ommatidia That Co-Expresses *rh3* and *rh4* in R7

**Figure 1:**
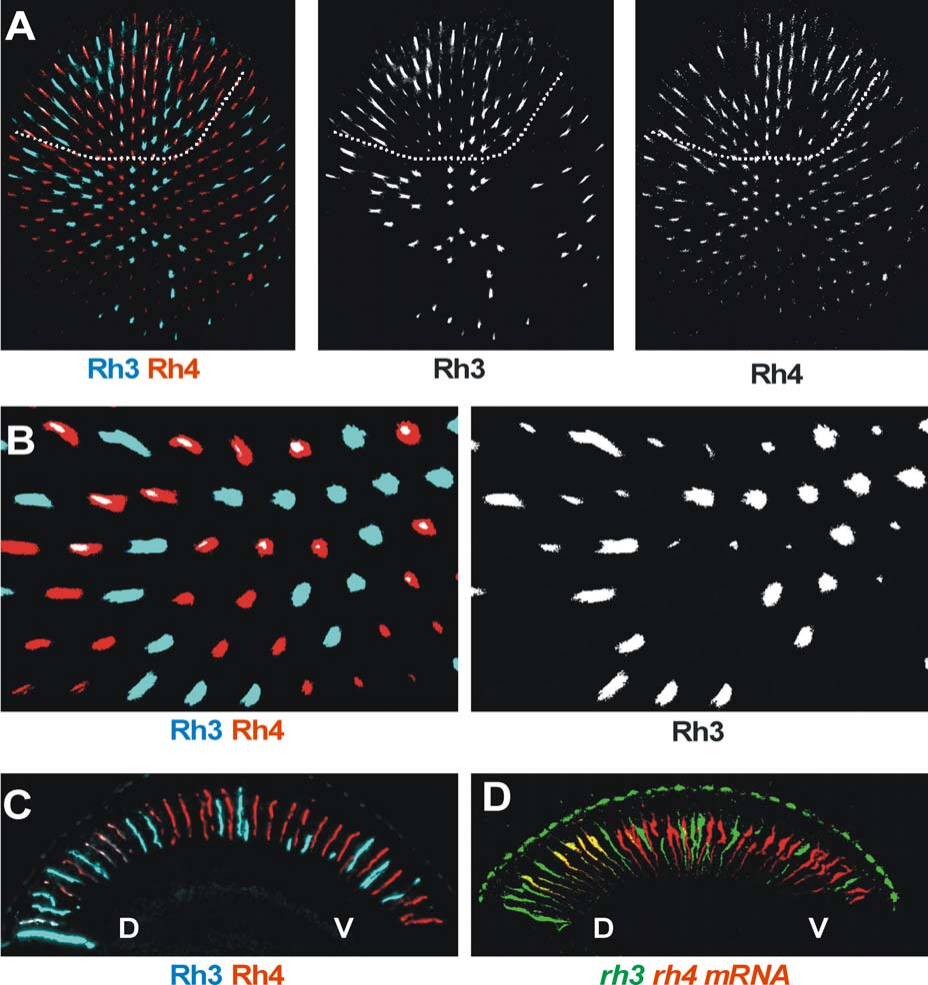
R7 Cells in the Dorsal Eye Co-Express *rh3* and *rh4* (A and B) Optical sections through a control eye stained for Rh3 (cyan) and Rh4 (red). Dorsal to the top. (A) R7 level: In ventral regions, R7 contain only Rh3 or Rh4. Dorsal R7 cells contain only Rh3, or Rh3 plus Rh4 (marked by dotted line). (B) Detailed view of the mid-dorsal region. The most dorsal yR7 cells co-express Rh3 and Rh4, while more ventral yR7 cells only express Rh4. (C) Transverse cryosection of a control eye stained for Rh3 (cyan) and Rh4 (red). Dorsal ommatidia clearly co-express Rh3 and Rh4. DRA ommatidia express Rh3 in both R7 and R8. D=dorsal, V=ventral. (D) In situ hybridization on a transverse section of a control eye with fluorescent probes for *rh3* (green) and *rh4* (red) mRNA. Co-expression of *rh3* and *rh4* mRNA is observed is observed in the dorsal retina. D = dorsal, V = ventral.

It is widely accepted that individual *Drosophila* photoreceptors express a single *rhodopsin* gene: *rh1* in R1–R6, *rh3* or *rh4* in R7 [15,31] (Figure 1A), and *rh5* or *rh6* in R8 [1,3]. However, careful examination of antibody stainings on whole-mounted retinas revealed a surprising exception to this rule: a fraction of R7 cells co-expresses both *rh3* and *rh4* (Figure 1A and 1B) in a region that starts near the dorsal edge of the eye, outside the DRA, and extends toward the equator, spanning approximately one-third of the eye at its maximum point (Figure 1A). This phenomenon is also clearly observed in cross-sections of the eye (Figure 1C). In the ventral region of the eye, Rh3 and Rh4 proteins are present at a high level in R7 cells and are never found in the same cell (Figure 1C, “V”). In contrast, all R7 cells located in the dorsal eye contain Rh3, either alone or in combination with Rh4 (p and y subtypes, respectively, see below) (Figure 1C, “D”). In R7 cells co-expressing *rh3* and *rh4*, the level of Rh3 protein is lower than in non–co-expressing cells (Figure 1A and 1B). Together, these data suggest that a subset of dorsal ommatidia induce *rh3* expression in *rh4-*expressing yR7 cells (Figure 1B). Rh3 and Rh4 colocalization was observed using different combinations of primary antibodies, indicating that this is not an artifact of a particular pair of antibodies (unpublished data), and co-expression is present in all wild-type backgrounds tested to date (*yw* and all other Gal4 and upstream activating sequence (UAS) lines used in this study), suggesting that this is a conserved feature of the *Drosophila* eye.

In our previous studies, we had detected expression of an *rh3* promoter fusion to a *green fluorescent protein* (*GFP*) reporter [32] in most ommatidia located in the dorsal eye. To distinguish whether the mutual exclusion or co-expression of *rhodopsins* in one cell results from transcriptional or post-transcriptional regulation, we performed double in situ hybridization to visualize *rh3* and *rh4* mRNA. In the ventral and central regions of the eye, *rh3* and *rh4* mRNA are present at high levels in a mutually exclusive manner (Figure 1D, “V”). However, in the dorsal eye, all R7 cells contain *rh3* mRNA, either alone or in combination with *rh4* mRNA (Figure 1D, “D”). Moreover, staining of *rh3*-lacZ reporter constructs consistently reveals expanded, weak *rh3* transcription in all ommatidia in the dorsal eye, whereas restricted expression to p ommatidia is observed in the remaining part of the retina (unpublished data and [33]). Together, these data indicate that there is localized transcriptional control of *rh3* and *rh4* that allows their co-expression in the dorsal retina.

**Figure 2:**
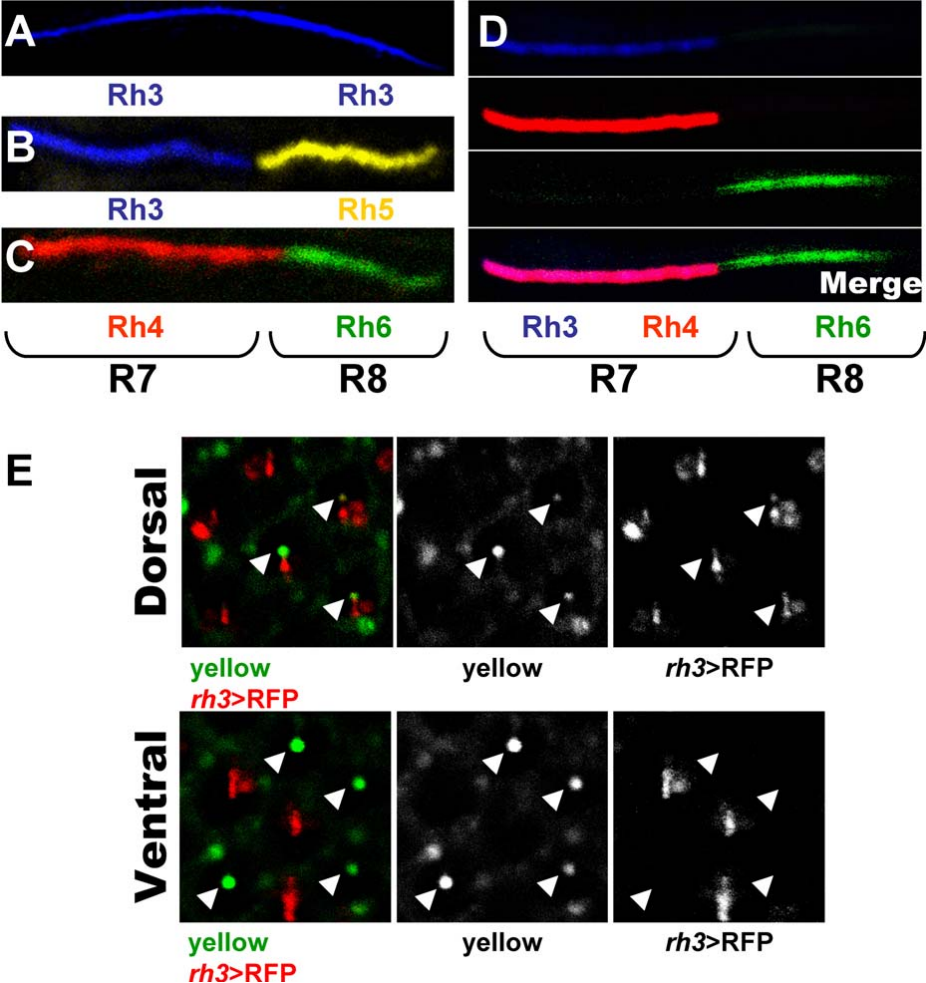
*rh3* and *rh4* Are Co-Expressed in yR7 Cells (A–D) Antibody staining of wild-type dissociated ommatidia. (A) DRA subtype: Rh3 (blue) is present in both R7 and R8. (B) p subtype: Pairing between Rh3 (blue) in R7 and Rh5 (yellow) in R8 is observed. (C) y subtype: Pairing between Rh4 (red) in R7 and Rh6 (green) in R8 is observed. (D) In the “dorsal” y subtype, Rh3 (blue) and Rh4 (red) are present in the same (pink) R7 and they are coupled with Rh6 (green) in R8. (E) Visualization of the “yellow” pigment (green) and *rh3>RFP* (red) using cornea neutralization technique of dorsal and ventral regions of the eye. The “yellow” pigment can be visualized due to its inherent fluorescence in the green channel. Arrowheads point to yR7 rhabdomeres. Note that, contrary to “yellow,” RFP is not restricted to the rhabdomere, allowing the whole cell to be visualized. Residual signal in pigment cells surrounding each ommatidium is also observed in the green channel.

We quantified the frequency of R7 cells co-expressing UV-opsins. In line with previous observations, antibody stainings on dissociated ommatidia identified the three previously described subtypes of ommatidia [1,3]: DRA ommatidia that contain Rh3 in both R7 and R8 (Figure 2A), p ommatidia that contain Rh3 and Rh5 (Figure 2B), and y ommatidia that contain Rh4 and Rh6 (Figure 2C) [16]*.* In addition, a small proportion (5.7%; 6/106) of all ommatidia (dorsal or ventral) express Rh3 in R7 (without Rh4) associated with Rh6 in R8. These likely correspond to the previously described rare Rh3/Rh6 “odd coupled” ommatidia where the signal from pR7 fails to induce *rh5* in R8 (unpublished data and [19,20,22]). However, we also identified a fourth subtype of R7 cells that contain both Rh3 and Rh4 in R7 cells (Figure 2D). These represent ∼10% of all ommatidia and are always coupled with Rh6-expressing R8 cells (Figure 2D). Stainings with anti-Rh4, anti-Rh5, and anti-Rh6 antibodies never revealed expression of Rh4 and Rh5 in the same ommatidium (0/200). yR7 cells contain a pigment that gave rise to their name (“yellow”) that is visible under the confocal microscope after neutralization of the cornea [17]. To further confirm that these co-expressing cells are yR7, we imaged the eyes of flies by confocal microscopy to visualize the “yellow” pigment as well as red fluorescent protein (RFP) controlled by the *rh3* promoter (*rh3*>RFP). As expected, “yellow” and *rh3*>RFP do not overlap in the ventral eye, because “yellow” marks yR7 cells and *rh3>*RFP labels pR7 cells. However, in the dorsal eye, “yellow” overlaps with *rh3*>RFP (Figure 2E).

We have thus identified a class of dorsal ommatidia that express both *rh3* and *rh4* in R7, and *rh6* in R8. These ommatidia represent a subset of y ommatidia that also express *rh3* in addition to the endogenous *rh4*. Ommatidia containing Rh3/Rh5 make up ∼30% of all ommatidia as evaluated by quantification of dissociated ommatidia (there is no Rh4/Rh5 coupling, and Rh5-positive ommatidia represent ∼30% [178/636] of all ommatidia). The remaining ∼70% of ommatidia express *rh6* (458/636). Stainings with anti-Rh3 and anti-Rh4 antibodies revealed that ∼30% of R7 express only *rh3* (85/273), ∼60% express only *rh4* (158/273), and ∼10% co-express *rh3* and *rh4* (30/273). Thus, the ∼70% *rh6*-expressing ommatidia can be divided into two subtypes: ∼60% of all ommatidia express *rh4/rh6* and ∼10% express (*rh3 + rh4)/rh6*, representing y ommatidia in the dorsal region of the eye.

### Genes of the *Iro-C* Complex Are Expressed Dorsally in the Retina

**Figure 3:**
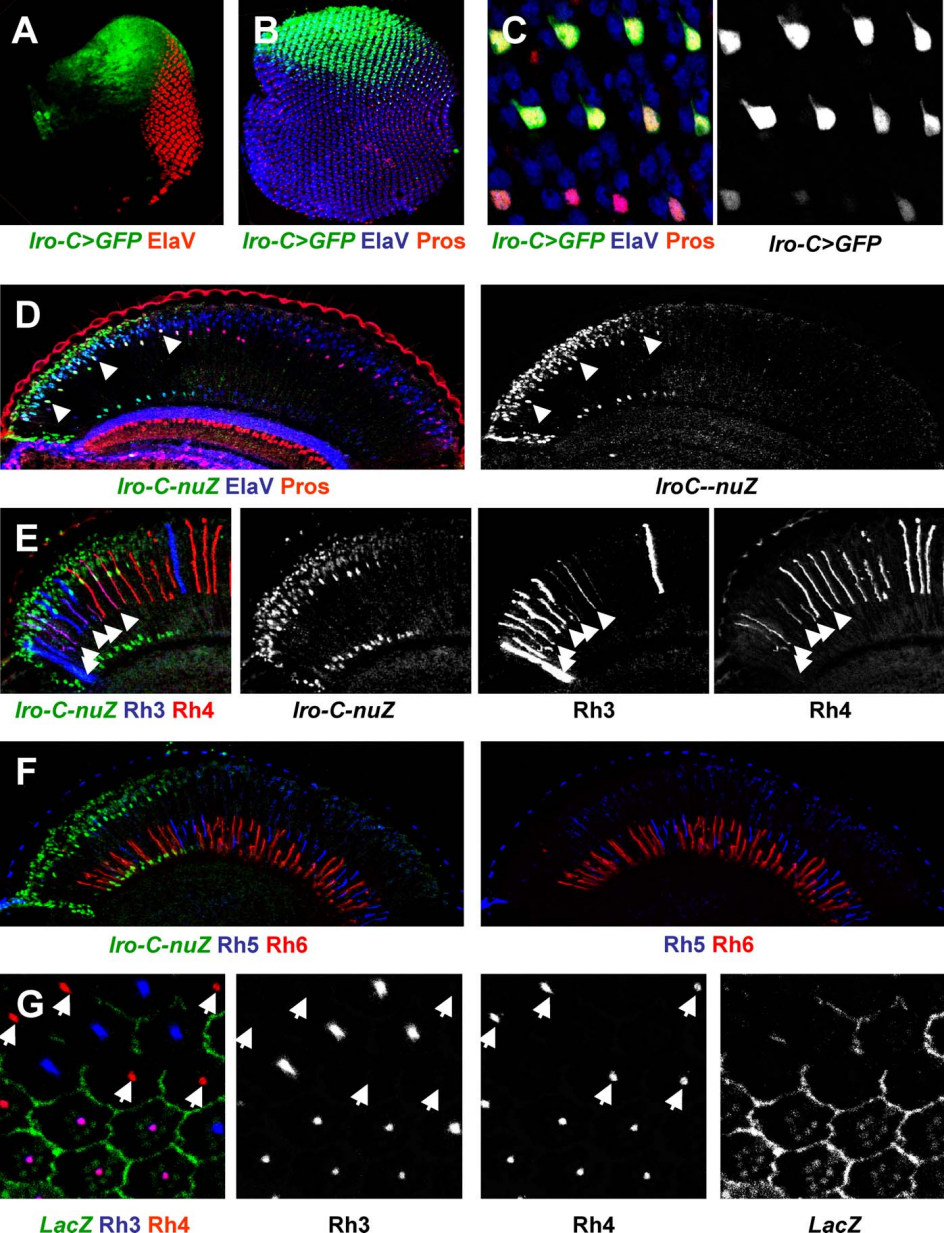
*Iro-C* Is Expressed in R7 Cells That Co-Express Rh3 and Rh4 (A) Larval eye imaginal disc stained for *Iro-C>GFP* (green). *Iro-C* is expressed in the dorsal half of the eye disc (top), whereas photoreceptor development is indicated by the neural marker ElaV (red). (B) Pupal eye (∼24 h APF): *Iro-C>GFP* (green) is expressed as a gradient in the dorsal (top) eye. The neural marker ElaV (blue) and the R7 marker Pros (red) are also shown. (C) Close up of the region of the eye where *Iro-C>GFP* expression (green) fades and can only be seen in R7 (marked by Pros (red)). (D) *Iro-C*-*nuZ* (green) expression in the adult eye. Expression in the dorsal eye (top) co-localizes with ElaV (blue) and Pros (red) in R7. Arrowheads indicate some R7 cell nuclei. (E) *Iro-C-nuZ* (green) is expressed in the adult eye in the dorsal region (left) where Rh3 (blue) and Rh4 (red) are co-expressed in R7 (arrowheads). (F) *Iro-C-nuZ* (green) expression with R8 specific markers Rh6 (red) and Rh5 (blue). (G) *Iro-C* mutant clones (marked by the absence of β-Gal; green) in the dorsal region of the eye stained with Rh3 (blue) and Rh4 (red). Mutant R7 do not co-express Rh3 and Rh4. Outside of the clone, all R7 cells that express Rh4 also express Rh3. White arrows mark *Iro-C* mutant R7 (β-Gal negative) that express only Rh4. Dorsal to the top.

*Iro-C* genes control dorsal identity during early eye development; therefore, we analyzed their expression in an effort to identify the determinants of this “dorsal” identity [28,30]. As mentioned earlier, these genes are expressed transiently during early larval stages of eye disc development (Figure 3A). *ara* and *caup* (but not *mirr*) are re-expressed in the adult in the dorsal retina. To perform a more detailed analysis of the *Iro-C* gene expression pattern, we used reporter lines (*Iro-C*-nuZ or *Iro-C*-Gal4), that are insertions in the *Iro-C* complex and are believed to reflect the expression of both *ara* and *caup* [21,27]. At 24 h after puparium formation (APF), the *Iro-C-*Gal4 reporter is highly expressed in all photoreceptors in the dorsal eye (Figure 3B). The level of expression gradually decreases toward the equator due to fewer and fewer cells per cluster that express the reporter. Ultimately, only R7 cells, identified with the R7-specific marker Prospero (Pros), express the reporter (Figure 3C). In the adult, the expression of the reporter persists in outer photoreceptors, as well as in R7 and R8 as previously shown [22]. This expression pattern correlates with the distribution of y ommatidia that co-express *rh3* and *rh4* in R7 and express *rh6* in R8 (Figure 3E and 3F) (see below for discussion). Thus, in the adult retina, the *Iro-C* genes *ara* and *caup* are specifically expressed in the region of the eye where there is co-expression of *rhodopsins.*

### *Iro-C* Genes Are Necessary for Co-Expression of *rh3* and *rh4* in R7

The similarity between the expression profile of the *Iro-C* genes *ara* and *caup* in the region of the eye where *rh3* and *rh4* are co-expressed suggested that these transcription factors regulate this newly defined subset of ommatidia. To test this hypothesis, we induced clones of cells that were mutant for *Iro-C* by using a deficiency that covers *ara* and *caup* and deletes most of the regulatory sequences of *mirr* [27,34]. Ventral clones are easily recovered but, as expected, they do not have a visible phenotype. While small dorsal clones do not produce a strong morphological phenotype, large clones often lead to the formation of ectopic eye tissue near the dorsal head cuticle, presumably because they create a new organizer between *Iro-C ^+^* and *Iro-C ^–^* cells (unpublished data)[28,30]. In the few dorsal mutant clones recovered, R7 cells co-express *rh3* and *rh4* in the surrounding heterozygous tissue, whereas in mutant tissue, R7 cells contain only Rh3 or Rh4 (Figure 3G). Thus, similar to the adult ventral eye where *Iro-C* is not expressed, dorsal *Iro-C* mutant R7 cells exclusively contain either Rh3 or Rh4. Therefore, *Iro-C* expression in the dorsal eye appears to be required for *rhodopsin* co-expression in R7 cells of dorsal y ommatidia*.*

**Figure 4:**
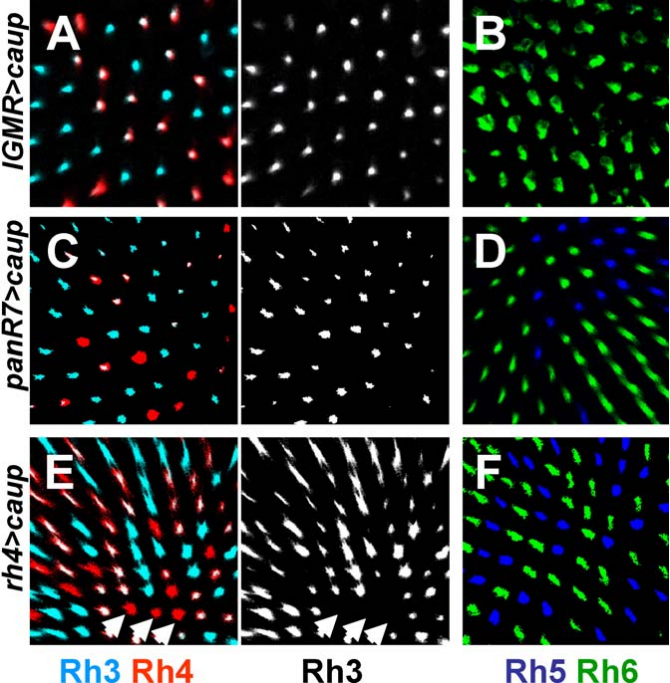
Over-Expression of *caup* Induces *rh3* and *rh4* Co-Expression (A) Optical section through the center (close to the equator, where there is no co-expression in control eyes, see Figure1) of eyes expressing *caup* in all photoreceptors under the *lGMR* promoter (*lGMR>caup*) and stained for Rh3 (cyan) and Rh4 (red). All yR7 cells containing Rh4 also contain Rh3 (white). pR7 cells only express Rh3 (cyan). (B) The same genotype stained for Rh5 (blue) and Rh6 (green) shows few R8 cells that contain Rh5; those are more frequent in ventral regions of the eye. (C) Similar staining as in (A) but *caup* is expressed in all R7 cells late during pupation (*PanR7>caup*). As in (A), most R7 cells that contain Rh4 also contain Rh3. pR7 only contain Rh3. (D) In the R8 layer, Rh5 (blue) and Rh6 (green) show a normal ratio. (E) Optical section through the center (equatorial) of the retina that expresses *caup* late during pupation only in yR7 (*rh4>caup*). Most yR7 cells contain both Rh3 and Rh4. Arrows indicate nontransformed ommatidia close to the equator. (F) In the R8 layer, Rh5 (blue) and Rh6 (green) show a normal ratio.

To study whether the *ara* and *caup* genes are sufficient to induce *rhodopsin* co-expression, we performed a series of mis-expression experiments. We observed essentially the same phenotype when over-expressing *ara* and/or *caup*, with the only difference being that the over-expression of both genes produces a more severe morphological phenotype than the expression of either one of them alone. We only show experiments using *UAS-caup,* but the same set of data is presented for *ara* in Figure S1. Because *mirr* is not expressed at this stage, we did not investigate its mis-expression phenotype. To mis-express *ara* and *caup* genes, we used the *long glass multiple reporter*-Gal4 (*lGMR*-Gal4*)* driver whose expression is restricted to all photoreceptors. *lGMR* expression starts during larval stages, after photoreceptors are specified at the morphogenetic furrow and is maintained throughout photoreceptor development and adulthood [19,35]. Over-expression of *caup* or *ara* at 25 °C leads to strong morphological defects in the eye, likely due to the prolonged expression of *Iro-C* genes when they are normally down-regulated during photoreceptor development. However, lowering Gal4 activity by raising flies at 18 °C induces robust *lGMR*>*caup–*dependent co-expression of *rh3* and *rh4* specifically in all yR7 cells (Figure 4A), whether ventral or dorsal. Importantly, *caup-*induced expansion of *rh3* in yR7 cells does not repress *rh4* expression. *lGMR>caup* over-expression does not induce ectopic expression of *rh3* in outer (R1–R6) or in R8 photoreceptors, and co-expression of *rh5* and *rh6* is not observed. However, *lGMR>caup* does increase to various degrees the proportion of *rh6*-expressing R8 cells with a corresponding decrease in *rh5*-expressing cells (Figure 4B). This expansion of Rh6 in R8 cells produces mis-coupling between R7 and R8 cells, resulting in an increase in ommatidia containing Rh3 in R7 and Rh6 in R8. Our interpretation is that, because *lGMR>Iro-C* produces morphological defects in the eye, the communication between R7 and R8 might be disrupted. In the absence of a signal from R7 to R8, most R8 cells express the default *rh6* (as in *sevenless* mutant eyes) [1,20].

We have previously shown that the decision between p and y fates is made during early pupation, when *ss* is activated in yR7 precursors, after *lGMR-*Gal4 starts to be expressed and long before *rhodopsins* are expressed [19]. To test whether *Iro-C* genes can cell-autonomously induce *rhodopsin* co-expression after the p versus y decision is made, *ara* and *caup* genes were expressed using a promoter that is expressed at late stages in development. *PanR7*-Gal4 is a combination of *rh3* and *rh4* promoters that is expressed in every R7 cell and in DRA R8 cells [19], starting at late pupal stages when *rhodopsin* expression starts [36]. Over-expression of *caup* using this late driver induces co-expression of *rh3* and *rh4* in the majority of R7 cells (Figure 4C), which are likely yR7 cells. To test whether a very late signal can induce co-expression in yR7, we expressed *Iro-C* genes using a *rh4*-Gal4 driver, which is only expressed in yR7 cells. This should allow R7 to be normally specified as yR7 and turn on *rh4*, which would then supply the *Iro-C* signal. Again, mis-expression of *caup* using this driver induces expression of *rh3* in most *rh4-*expressing cells (Figure 4E)*.* The phenotype is stronger in the central or more-dorsal areas than in ventral regions where this driver is not able to transform all yR7 cells, because it might lack the strength of the *PanR7* driver. Together, these results suggest that there is an endogenous sub-threshold level of *Iro-C* in the dorsal eye close to the equator that is not sufficient to induce co-expression of *rh3* and *rh4* in a wild-type situation. The *PanR7-* and *rh4*-Gal4 drivers must only add limited amount of *ara* or *caup*, or provide it late, such that not all yR7 cells co-express. Neither *PanR7*- nor *rh4-Gal4* drivers induce phenotypes in R8 cells (31.7% and 33% of *rh5* expression, respectively) (Figure 4D and 4F), suggesting that, as expected, the early decision between p and y fates is not affected. In addition, the expression of *caup* only in R8 with *rh5*- and *rh6*-Gal4 drivers does not produce a visible phenotype (Figure S1G). Therefore, the presence of the Caup or Ara transcription factors in yR7 cells, even very late in development, instructs them to co-express *rh3* and *rh4*.

## Discussion

The *Drosophila* retina presents a stochastic distribution of ommatidial subclasses. As described before, in ∼30% of ommatidia, R7 express *rh3* and R8 express *rh5*, whereas in the remaining ∼70%, R7 express *rh4* and R8 express *rh6* [1,16,37]*.* These numbers are correct if we consider only R8 *rhodopsin* expression. However, the data presented here indicate that, if one considers the distribution of R7 *rhodopsins*, the y ommatidial subtype should be divided into two subpopulations: the “classical y” subtype that expresses only *rh4* in R7 and *rh6* in R8; and the “dorsal y” subtype that expresses both *rh3* and *rh4* in R7 and *rh6* in R8. Thus, *Drosophila* contain four (or even five, if we consider the “odd coupled” ommatidia) rather than the three classes that were previously described: DRA, p, y, and “dorsal y” ommatidia.

The expression of *ara* and *caup* allows co-expression of *rhodopsins* in R7 cells by inducing the expression of *rh3* in *rh4*-expressing cells. Although *Iro-C* gene products could activate the *rh3* promoter directly, it should be noted that they are expressed in all photoreceptors in the dorsal eye, but *rh3* is only induced in *rh4*-expressing R7. In addition, over-expression of *Iro-C* in all photoreceptors using *lGMR*-Gal4 only induces *rh3* in R7, and not in other photoreceptors (Figure 3A).

**Figure 5:**
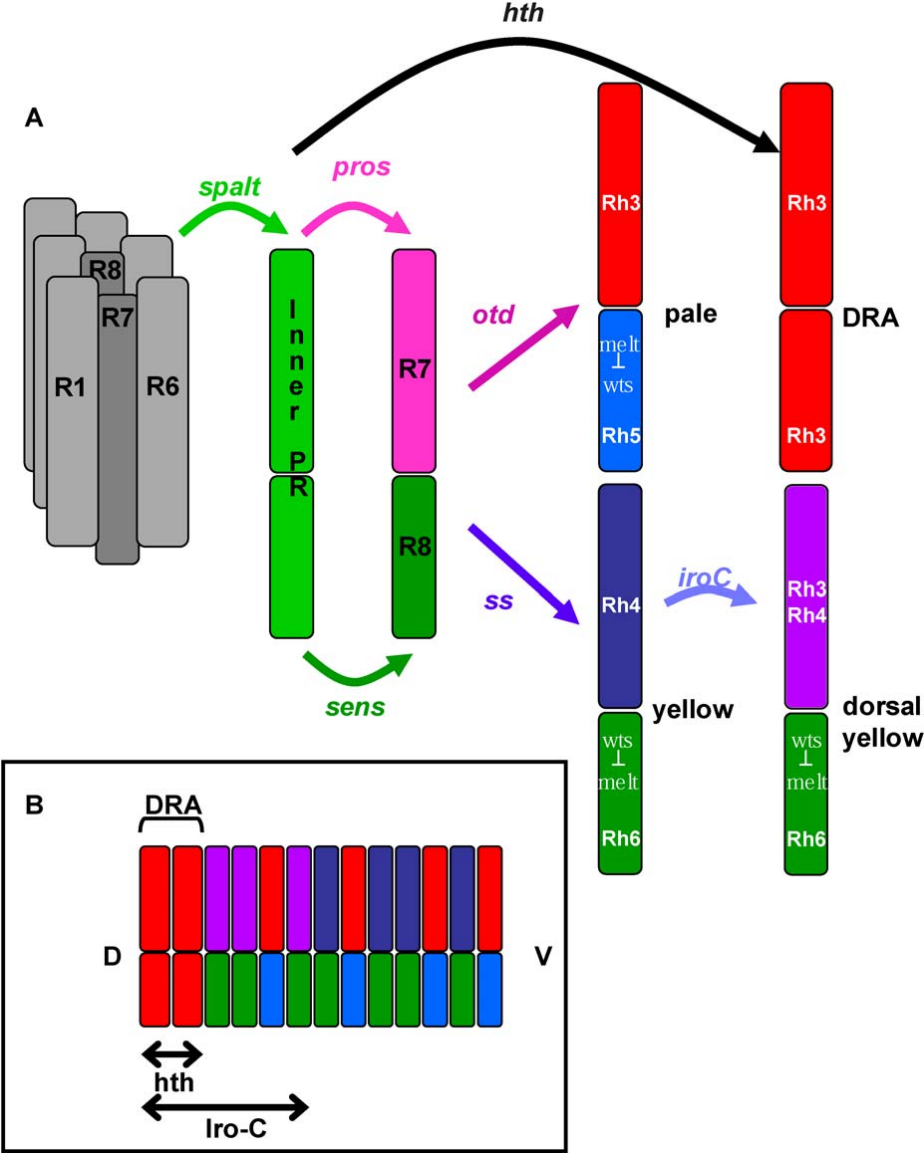
Generation of Retinal Subtypes (A) During development, the first cell-fate decision divides photoreceptors into two different subtypes, inner (R7 and R8) and outer (R1–R6). This separation is mediated by the *spalt* (*sal*) genes in inner photoreceptors. After the acquisition of a generic ‘inner' fate, *prospero* (*pros*) is expressed in R7 and directs cells towards the R7 fate. *senseless* (*sens*) plays a parallel role in R8 cells and directs cells towards the R8 fate. The dorsal most row of ommatidia is then specified as DRA by the expression of *homothorax* (*hth*) and becomes a polarized light detector. The main part of the retina is then patterned into the y and p subtypes. *orthodenticle* (*otd*) is expressed in all photoreceptors and is required for the direct activation of *rh3* and *rh5* in the p subtype. Expression of *spineless* (*ss*) in a subset of R7 cells then generates the y subtype. *Iro-C* genes *ara* and *caup* then act in yR7 cells and allow the co-expression of *rh3* and *rh4*. (B) Distribution of ommatidial subtypes along the dorso-ventral axis of the retina. DRA photoreceptors are located most dorsally. The dorsal eye contains either pure p or dorsal y ommatidia generated in response to *Iro-C* that extends through the dorsal eye. The central and ventral regions of the eye contain true p and y ommatidia.

During development, photoreceptors are subdivided first into two different subtypes, inner (R7 and R8) and outer (R1–R6) by the expression of the two transcription factors encoded by the *spalt* (*sal*) complex in inner photoreceptors (Figure 5A). After photoreceptors acquire a generic “inner” fate, *prospero* is expressed in R7 and directs it away from the R8 fate and toward an R7 fate. Similarly, *senseless* plays a parallel role in R8 cells to prevent R7 differentiation [38]. At this stage, R7 and R8 are specified as photoreceptors, but they are not patterned in terms of *rhodopsin* expression. The dorsal-most row of ommatidia is then specified as DRA by the expression of *homothorax* (*hth*), which differentiates them from the rest of the retina to become polarized light detectors (Figure 5A and 5B). The main part of the retina is then patterned into the y and p subtypes by the expression of *ss* during pupation in a subset of R7 cells (Figure 4A). R7 cells that do not express *ss* become pR7 cells by default [19]*. rh3* is activated by *orthodenticle* (*otd*), which is present in all photoreceptors, and thus its activity must be actively repressed in yR7 cells*. ara* and *caup* might be the signal in yR7 cells that allows the expression of the default state *rh3* and breaks the mutual exclusion pathway between *rhodopsins* (Figure 4A and 4B)*.* R8 cells that are located below dorsal yR7 cells cannot co-express *rhodopsins* because a bi-stable loop between *warts* and *melted* does not allow an ambiguous choice between the *rh5* and *rh6* fates after the decision is made [39].

### Functional Consequences of Sensory Receptor Co-Expression

The co-expression of *rhodopsins* in R7 is restricted to the dorsal eye, which faces the sky. The biological significance of these particular ommatidia in *Drosophila* is not known. The *Drosophila* “dorsal y” ommatidia that contain both UV-Rh3 and UV-Rh4 in R7 and green-absorbing Rh6 in R8 provide a unique configuration to measure the ratio between UV and long wavelengths: They contain two UV opsins in R7, providing broad UV sensitivity that is expanded toward shorter wavelengths by Rh3, along with a blue-filtering pigment that prevents short wavelengths to penetrate the R8 layer containing the green-absorbing Rh6 [8]. These ommatidia might be used to discriminate between the “solar” and “antisolar” halves of the sky, necessary to navigate in the correct direction [40].

Although the exclusion of sensory receptors is a general rule, co-expression to achieve a novel sensitivity might be used in special cases when the expression of a single receptor is not sufficient to confer high enough sensitivity. Although the mouse retina is dominated by rods, it also contains cone cells. The majority of these cone cells co-express both S (blue) and M (green) opsins [41]. Presumably, mice live in a dark environment and are mostly color blind; the co-expression might be useful for optimal utilization of cones. The eye of butterflies also displays co-expression of two *rhodopsins* in several of their photoreceptors, perhaps to expand the spectrum of sensitivity of photoreceptors in species that do not have a *rhodopsin* with broad absorption spectrum such as Rh1, which is unique to Diptera [42–44].

Vertebrate olfactory neurons also express only one olfactory receptor gene per olfactory receptor neuron, and a direct feedback from the expressed receptor molecule has been proposed to ensure that this rule is stringently applied [45–47]. However, it cannot be excluded that two olfactory receptor genes are co-expressed, because their large number prevents comprehensive expression studies. Indeed, in *Drosophila*, a striking example of co-expression of two chemosensory receptors that mediate sensitivity to CO_2_ was recently described for the olfactory system [48]. The expression of each receptor is not sufficient to confer olfactory CO_2_-chemosensation on its own, but their combined expression does. Therefore, the addition of multiple receptors might not only increase the receptive spectrum of cells, but might also confer sensitivity to new stimuli. In the CO_2_ sensitivity case, the co-expression is crucial for the fly to detect a repellent smell that indicates danger. Thus, precise regulation of receptor co-expression must be achieved.

### Stochastic Versus Regionalized Specification in the Retinal Mosaic

The spatial specialization induced by *Iro-C* genes in the fly retina is not the only example where regionalized specification occurs within sensory systems. For example, in the “love spot” of the housefly *Musca*, the antero-dorsal region of the male eye has presumably lost color vision, because R7 cells are transformed into motion detecting outer photoreceptors that express Rh1 [49]. The human eye also has geographic specialization: the center of the eye (fovea) contains exclusively cones that are involved both in acute and color vision in bright light. The periphery of the eye is mostly composed of rods and is involved in dim light vision (reviewed in [50]). The mouse olfactory system also exhibits specialization where the main olfactory epithelium that is responsible for detection of general odorants is separated from the vomeronasal organ that is involved in pheromone detection [51]. *Drosophila* also has two olfactory organs, the antenna and the maxillary palps, which express different sets of olfactory receptors and are likely involved in the detection of different types of odors [52].

### Conserved Role for *Iro-C* in Sensory Receptor Co-Expression?

*Iro-C* genes may not only be responsible for relieving the “one receptor–one neuron” constraint in the *Drosophila* eye, but may also allow receptor co-expression elsewhere. For instance, members of the orthologous family, the *Irx* genes, are expressed in mouse photoreceptors where opsin co-expression is observed [53–55]. Although the terminal differentiation of bipolar cells is affected in mice with mutant *Irx5* [54], it will be of interest to study cone *opsin* expression in this and other *Irx* mutants to test whether these genes are also involved in the co-expression of opsins.

Mouse olfactory neurons do not express *Irx5* or *Irx6* [53], and they do not express more than one olfactory receptor gene [56]. In contrast, recent comprehensive studies in the *Drosophila* antenna and maxillary palp have identified a subgroup of olfactory receptor neurons that co-express two divergent receptors [57,58]. Interestingly, cells that co-express different olfactory receptor genes are the only neurons that express *Iro-C* genes in the maxillary palp (EOM, AC, and CD; unpublished observations). Unfortunately, the loss of *Iro-C* function in this tissue leads to re-specification of these neurons toward other non-neuronal fates (EOM, AC, and CD; unpublished observations), preventing us from further testing the involvement of *Iro-C* genes in the lack of exclusion.

Genes directly controlled by *Iro-C* transcription factors are still elusive. Binding sites for Mirr that presumably mediate repression of *fringe* in the dorsal eye disc were recently described [59]. The identification of target genes of the *Iro/Irx* family might shed some light on the regulation of the pathway that maintains mutual exclusion of sensory receptors.

## Materials and Methods

### *Drosophila* strains and crosses.

Flies were raised on standard corn meal–molasses–agar medium and grown at room temperature (24 ± 1 °C) unless otherwise noted. *y^1^w^67^* flies were used as control for Rhodopsin expression. As the red color of adult eyes interferes with fluorescent immunostainings, the eyes were rendered white by using an RNAi construct against the *white* gene [60] when a *white* marker gene was introduced in the genetic background by P-element transgenes. *lGMR*-Gal4 was produced by a pentamerized Glass binding site [22], UAS-*ara* and *caup* were gifts from J. Modolell. iro^rF209^-PZ and Df(3L)iro^DFM3^ were obtained from the Bloomington Stock Center. *Iro-C*-Gal4 was created by replacing the P element in iro^rF209^-PZ with one containing Gal4. *rh3-, rh4*, and *PanR7-*Gal4 drivers were described in [19]. To visualize “yellow” and *rh3* expression with a reporter, we used flies containing *rh3-*LexA and lexAop-RFP. Clones were generated using the standard FLP/FRT technique.

### Antibodies.

Antibodies and dilutions used were as follows: mouse anti-Rh3 and anti-Rh4 (1:100) and mouse anti-Rh5 (1:50) (gift from S Britt, University of Colorado); rabbit anti-Rh4 (1:400) (gift from C. Zuker, University of California San Diego); rabbit anti-Rh6 (1:5000); rabbit anti-βGal (1:5000) (Cappel); mouse anti-βGal (1:500) (Promega); mouse anti-pros (1:50) (Developmental Studies Hybridoma Bank); rat anti-ElaV (1:10) (DSHB); and rabbit anti-GFP (1:800) (Biogenesis). Chicken anti-Rh3 was from [61]. All secondary antibodies were Alexa-conjugated (1:800) (Molecular Probes). Throughout the paper, Rh3 and Rh4 were stained using antibodies generated in mouse and rabbit, respectively, because they are significantly better that the other two.

### Antibody stainings for larval and pupal retinas.

Antibody stainings for larval and pupal retinas were essentially the same except for the collection of tissue. The protocols merge after the fixation step. Cerebral complexes of late third instar larvae were dissected in phosphate buffered saline (PBS) (1×) and fixed in PBS + 4% paraformaldehyde for 20 min at room temperature (RT). Pupal cases were collected at 24 h after puparium formation at 25 °C and the head was dissected in ice cold PBS (1x). Several eye-brain complexes were extracted by gentle pipetting and collected in PBS (1×) on ice. After 20 min fixation using PBS (1×) + 4% formaldehyde at RT, the samples were washed four times with PBS + 0.1% Triton-X-100 (PBT). The first antibody was added overnight at 4 °C. After four washes with PBT, the secondary antibody was added for at least 2 h at RT. After another four washes in PBT, each retina was separated from the brain by using two tungsten needles and then mounted flat in Vectashield (Vector Laboratories).

### Antibody stainings for frozen fly head sections.

10-μm horizontal eye sections were produced using a cryostat (Zeiss) and deposited on Superfrost PLUS slides (Fisher). The slides were then fixed 15 min in PBS (1×) + 4% formaldehyde. After four washes with PBT, the first antibody was added overnight at 4 °C. After four washes with PBT, the secondary antibody was added for at least 2 h at RT. After four washes with PBT, the slides were mounted in Aquamount.

### Antibody stainings for adult whole-mounted retinas.

Adult retinas were dissected out and after a rinse with PBS (1×), they were fixed for 15 min with 4% formaldehyde at RT. After three washes in PBT, the retinas were incubated with the primary antibodies diluted in BNT (PBS, 0.1% BSA, 0.1% Tween-20, 250 mM NaCl) overnight at 4 °C. After two rinses and a 30 min wash with PBT, the retinas were incubated with secondary antibodies for 2–4 hours at RT. Two quick rinses with PBT were followed by an overnight wash at 4 °C. Retinas were cleaned of any remaining cuticle and mounted in Vectashield.

### Antibody stainings in dissociated ommatidia.

The retina of 3–5 dissected eyes were removed from the cornea and dissociated on a slide using dissection needles in a drop of PBS. After the samples dried at RT, they were fixed with 4% formaldehyde and staining was carried out as for frozen sections.

### In situ hybridization for cryosectioned adult retinas.

Adult retinas were dissected as described for antibody stainings. Dissected retinas were mounted on Superfrost PLUS slides (Fisher) and dried for 2 h at 65 °C. After fixation for 15 min with 4% paraformaldehyde, the slides were washed in PBS, treated with Proteinase K for 5 min at 37 °C and refixed for 10 min. Following a short PBS wash, the slides were treated with 0.2 M HCl for 10 min, washed in PBS, and acetylated with 0.1 M triethanolamine. Retinas were hybridized overnight at 65 °C with 100 μl hybridization buffer (50% formamide, 5× SSC, 5× Denhardt's, 250 μg/ml yeast tRNA, 500 μg/ml herring sperm DNA, 50 μg/ml heparin, 2.5 mM EDTA, 0.1% Tween-20, 0.25% CHAPS) containing a digoxygenin-labeled *rh3* probe and a fluorescein-labeled *rh4* probe. After a series of washes in 5× SSC; 50% formamide, 2× SSC; 2× SSC; 0.2× SSC, and 0.1× SSC (5, 30, 20, 20, and 20 min, respectively), the *rh3* probe was detected using HNPP/Fast Red (Roche) and the *rh4* probe was detected using the TSA Biotin System (Perkin Elmer) and streptavidin-Alexa488 according to the manufacturers suggestions.

### Neutralization of the cornea.

Anesthetized flies were fixed to a Petri dish using nail polish. Then, flies were submerged in water and visualized using a 20× water immersion lens. To visualize “yellow”, FITC settings were used [31].

## Supporting Information

Figure S1 Over-Expression of *ara* in R7 and *caup* in R8 (A) Optical section through the center (equatorial) of eyes expressing *ara* in all photoreceptors under the *lGMR* promoter (*lGMR>ara*) and stained for Rh3 (cyan) and Rh4 (red). Almost all yR7 cells containing Rh4 also contain Rh3 (white). pR7 cells only contain Rh3.
(B) Fly eyes of the same genotype stained for Rh5 (blue) and Rh6 (green) show few R8 cells containing Rh5; those are more frequent in ventral regions of the eye.
(C) Similar staining as in (A), but *ara* is expressed in all R7 cells late during pupation (*PanR7>ara*). As in (C), most R7 cells that contain Rh4 also contain Rh3. pR7 only contain Rh3.
(D) In the R8 layer, Rh5 (blue) and Rh6 (green) show a normal ratio.
(E) Optical section through the center (equator) of the retina that expresses *ara* late during pupation only in yR7 (*rh4>ara*). Most yR7 cells contain both Rh3 and Rh4. Arrows indicate nontransformed ommatidia close to the equator.
(F) In the R8 layer, Rh5 (blue) and Rh6 (green) show a normal ratio.
(G) Over-expression of *caup* using R8 specific drivers (*rh5+rh6>caup*) does not induce any phenotype.
(6.05 MB PDF)

## Abbreviations

PBS: phosphate buffered saline
DRA: dorsal rim area
*lGMR*: *long glass multiple reporter*
p: pale type
Rh: rhodopsin
RT: room temperature
UV: ultraviolet
y: yellow type
