# Supplementary figures and images for: Iroquois Complex Genes Induce Co-Expression of rhodopsins in Drosophila
Source: PLoS Biol. 2008 Apr 22;6(4):e97. doi: 10.1371/journal.pbio.0060097 (PMC2323304; doi:10.1371/journal.pbio.0060097)

*IGMR>ara*

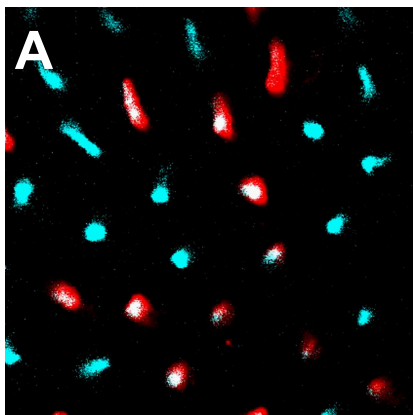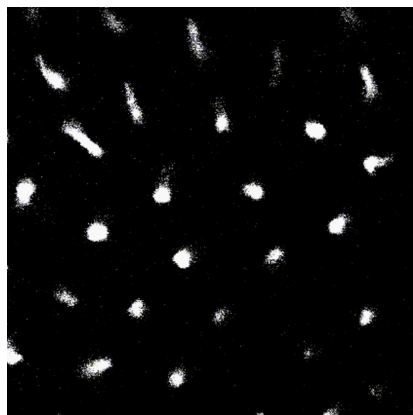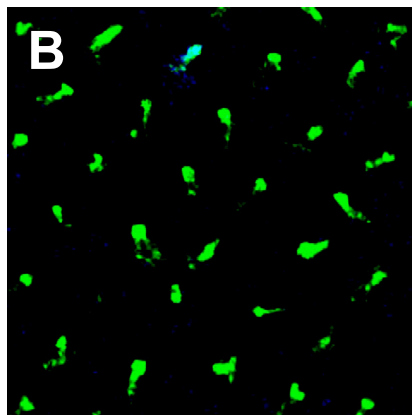

*panR7>ara*

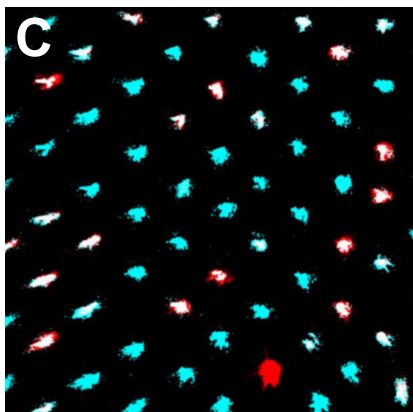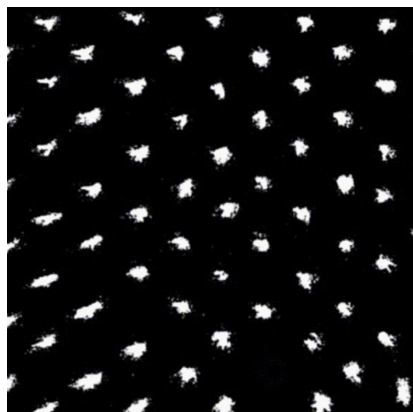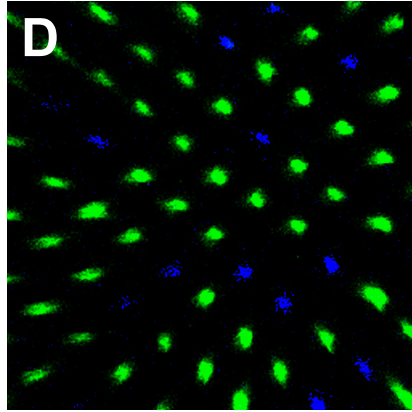

*rh4>ara*

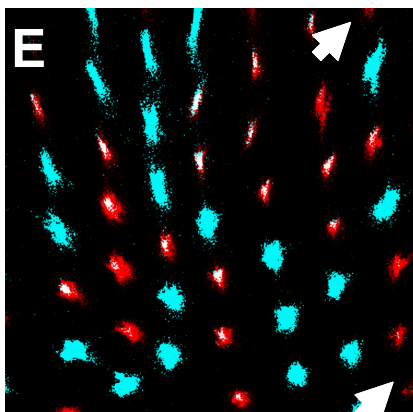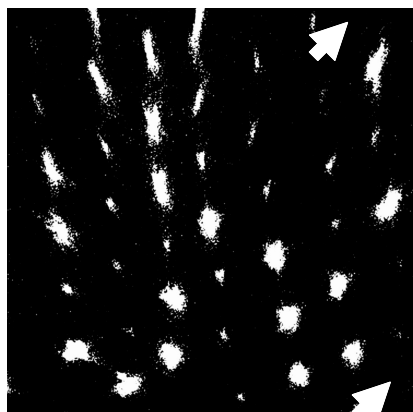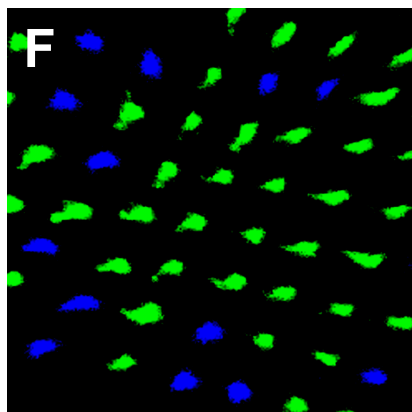

Rh3 Rh4

Rh3

Rh5 Rh6

*rh5+rh6>caup*

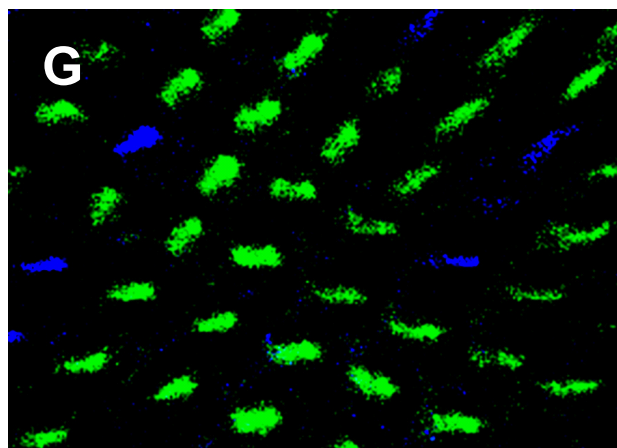

Rh5 Rh6

Supplement: Figure S1 — (A) Optical section through the center (equatorial) of eyes expressing ara in all photoreceptors under the lGMR promoter (lGMR>ara) and stained for Rh3 (cyan) and Rh4 (red). Almost all yR7 cells containing Rh4 also contain Rh3 (white). pR7 cells only contain Rh3. (B) Fly eyes of the same genotype stained for Rh5 (blue) and Rh6 (green) show few R8 cells containing Rh5; those are more frequent in ventral regions of the eye. (C) Similar staining as in (A), but ara is expressed in all R7 cells late during pupation (PanR7>ara). As in (C), most R7 cells that contain Rh4 also contain Rh3. pR7 only contain Rh3. (D) In the R8 layer, Rh5 (blue) and Rh6 (green) show a normal ratio. (E) Optical section through the center (equator) of the retina that expresses ara late during pupation only in yR7 (rh4>ara). Most yR7 cells contain both Rh3 and Rh4. Arrows indicate nontransformed ommatidia close to the equator. (F) In the R8 layer, Rh5 (blue) and Rh6 (green) show a normal ratio. (G) Over-expression of caup using R8 specific drivers (rh5+rh6>caup) does not induce any phenotype. (6.05 MB PDF) [file pbio.0060097.sg001.pdf]
